# Health insurance coverage in Ethiopia: financial protection in the Era of sustainable cevelopment goals (SDGs)

**DOI:** 10.1186/s13561-022-00389-5

**Published:** 2022-08-03

**Authors:** Bedasa Taye Merga, Bikila Balis, Habtamu Bekele, Gelana Fekadu

**Affiliations:** 1 School of Public Health, College of Health and Medical Sciences, Haramaya University, Harar, Ethiopia; 2 School of Nursing and Midwifery, College of Health and Medical Sciences, Haramaya University, Harar, Ethiopia

**Keywords:** Insurance, Healthcare financing, Ethiopia, DHS

## Abstract

**Background:**

Health insurance is among the healthcare financing reforms proposed to increase the available healthcare resources and to decrease the risk of household financial crisis. Recently, Ethiopia has been implementing community-based health insurance which mainly targets the very large rural agricultural sector and small and informal sector in urban settings. Therefore, this study was aimed to assess the coverage of health insurance and its determinants in Ethiopia.

**Methods:**

Data were extracted from the 2019 mini Ethiopian Demographic and Health Survey (EDHS) to assess determinants of health insurance coverage in Ethiopia. The analysis included a weighted sample of 8663 respondents. Multivariable logistic regression analysis was conducted and the results were presented as adjusted odds ratio (AOR) at 95% confidence interval (CI), statistical significance was declared at a *p*-value < 0.05 in all analyses.

**Results:**

The health insurance coverage in Ethiopia was 28.1% (95%CI: 27.2%, 29%). Administration regions (Tigray: AOR = 16.9, 95%CI: 5.53, 51.59, Amhara: AOR = 25.8, 95%CI: 8.52, 78.02, Oromia, AOR = 4.27, 95%CI: 1.41, 12.92, Southern Nations, Nationalities and Peoples region, AOR = 4.06, 95%CI: 1.34, 12.32, Addis Ababa, AOR = 4.65, 95%CI: 1.46, 14.78), place of residence (rural, AOR = 1.38, 95%CI: 1.17, 1.63), sex of household head (male; AOR = 1.23, 95%CI: 1.07, 1.41), wealth index (middle, AOR = 1.75, 95%CI: 1.46, 2.09, richer, AOR = 1.86, 95%CI: 1.55, 2.24), family size (≥ 5 members, AOR = 1.17, 95%CI: 1.03, 1.33), having under-five children (AOR = 1.22, 95%CI: 1.076, 1.38), and age of household head (31–40 years, AOR = 1.71, 95%CI: 1.45, 2.01, 41–64 years, AOR = 2.49, 95%CI: 2.12, 2.92, 65 + years, AOR = 2.43, 95%CI: 2.01, 2.93) were factors associated with health insurance coverage.

**Conclusions:**

Less than one-third of Ethiopians were covered by health insurance. Socio-economic factors and demographic factors were found to associate with health insurance coverage in Ethiopia. Therefore, enhancing health insurance coverage through contextualized implementation strategies would be emphasized.

**Supplementary Information:**

The online version contains supplementary material available at 10.1186/s13561-022-00389-5.

## Introduction

The sustainable development goal for health (SDG3) envisioned ensuring healthy lives and promoting well-being for all at all ages [1]. SDG3 also includes a sub-target (3.8) on achieving universal health coverage, including financial risk protection and access to quality, essential health services. Nevertheless, paying for healthcare has financial consequences for households. Survey data from 133 countries suggest that about 808 million people experienced catastrophic health expenditures in 2010 [2], while approximately 97 million people suffered impoverishment due to health spending [3], and others went without seeking healthcare altogether due to a lack of affordability and thus suffered from important unmet medical need.

Financial risk protection from the costs of illness is among the ultimate goals of health care systems. As the cost of healthcare is ever increasing, most of the countries are facing difficulties in meeting the health needs of their citizens [4]. Direct out of pocket payment for healthcare involves no risk pooling and is not effective way of covering healthcare costs. Besides, direct out of pocket payment is found to be one of the deterrents of healthcare service consumption in low and middle income countries (LMICs) [5].

Health insurance is among the healthcare financing reforms proposed to increase the available healthcare resources and decrease the risk of household financial crisis [6]. Health insurance has been endorsed in LMICs to improve access to healthcare services because it avoids direct payments of fees by patients and spread the financial risk among all the insured members [7, 8]. Community-based health insurance (CBHI) is one of the health insurance schemes mainly proposed with aim of reducing out of pocket payment, particularly in areas where many people engage in informal workers and rural residence [9].

Since 2011, Ethiopia has been implementing CBHI and proposed social health insurance that covers those engaged in formal sectors [10]. Currently, health insurance in Ethiopia targeted the rural areas and citizens mainly recruited in informal sectors such as agriculture. Its expansion in the recent time has brought a significant improvements in healthcare utilizations and financial risk protection [10].

Cognizant of its significance in relieving the financial crisis and its proven importance in improving resource mobilization for healthcare [11], the health insurance coverage in Ethiopia remained low. In 2019, in Ethiopia health insurance coverage was 28% [12], which was far below the national targets by 2020 [13]. Besides the coverage varies across administrative regions and other socio-demographic factors [12].

Synthesis of studies conducted in different parts of the countries shows that community-based health insurance coverage is influenced by individual health seeking behavior [14], socio-economic status of the household, place of residences [15]. In order to increase enrollment into health insurance, identifying factors associated with it has utmost significance.

In Ethiopia, although different studies have been conducted assessing enrollment into health insurance, most of these studies are pocket-sized covering small geographic areas with small sample size. Therefore, this study is aimed to assess the coverage of health insurance and its determinants among Ethiopian households using national data from EDHS 2019. Ethiopian DHS is a nationwide survey that includes both rural and urban residents from nine regional states and two cities administrations of the country.

Given the national and global targets of universal health coverage and financial protection, this study has paramount importance in providing factual insights regarding the current status of health insurance coverage and its associated factors in Ethiopia.

## Methods

### Data sources

This study utilized data from the 2019 Ethiopia Mini Demographic and Health Survey (EMDHS). The Ethiopian Public Health Institute conducted the survey in collaboration with the Central Statistical Agency (CSA) and the Federal Ministry of Health (FMoH), with technical assistance from ICF and financial as well as technical support from development partners [16].

The survey was conducted from March 21, 2019, to June 28, 2019, based on a nationally representative sample that provided estimates at the national and regional levels and for urban and rural areas. The survey interviewed 8,855 women of reproductive age (age 15–49) from a nationally representative sample of 8,663 households. This study included a total of 8,663 households from nine regional states and two city administrations. Details about the DHS sampling techniques and sample size are available at http://www.dhsprogram.com/. The EDHS research protocol complies with the National Health Research Ethics Committee and Institutional Review Board guidelines.

### Study variables

Dependent variable: health insurance coverage which was assessed by asking whether the household is enrolled into any health insurance schemes.

Independent variables: Administrative regions (Tigray, Afar, Amhara, Oromia, Somali, Benishangul-Gumuz, SNNP, Gambela, Harari, Addis Ababa, and Dire Dawa), Place of residence (urban, rural), Sex of household head( male, female), Wealth index (poorest, poorer, middle, richer, richest), Family size( categorized as ≤ 5 members, > 5 members), Under-five age children (categorized as Yes, No), Age of the household head (recorded as continuous and we categorized as 15- 30 years, 31–40 years, 41–64 years, 65 + years), Health facility visits (categorized as only one visit, two or more visits).

### Data management and statistical analysis

Extracted data were weighted so that the sample was representative of respondents in 2019 mini EDHS. Analyses were performed using STATA version 14. To assess the association between socio-demographic characteristics and other explanatory variables, and health insurance coverage, a logistic regression model was employed.

First, each variable was entered into a binary logistic regression model. Second, variables which were significant at a p-value of less than or equals 0.25 were fitted into a multivariable logistic regression model to identify independent factors of health insurance coverage in Ethiopia. Statistical significance was declared at a *p*-value < 0.05 in all analyses. The results from the logistic regression analyses are presented as adjusted odds ratios (OR) with 95% confidence intervals (CIs).

## Results

### Socio-demographic characteristics of study participants

The study included a total of 8663 households from the nine administrative regions and two city administrations. Majority (69.25%) of the study participants were from rural residence. The mean age of the study participants was 43.0 4 (± 16.49) years. Only 10.9% of the participants had two and more healthcare facility visits per year. About half of the (49.3%) of the households had under-five children Table 1.

**Table 1:** Socio-demographic characteristics of study participants, Ethiopia, 2019 (*n* = 8663)

Variables	Categories	Frequency	Percent (%)
**Administrative regions**	Tigray	585	6.75
	Afar	88	1.01
	Amhara	2,109	24.35
	Oromia	3,207	37.02
	Somali	419	4.83
	Benishangul-Gumuz	94	1.08
	SNNP	1,668	19.25
	Gambela	35	0.41
	Harari	25	0.29
	Addis Ababa	376	4.34
	Dire Dawa	57	0.66
**Place of residence**	Urban	2,664	30.75
	Rural	5,999	69.25
**Sex of household head**	Male	6,751	77.93
	Female	1,912	22.07
**Wealth index**	Poorest	1,498	17.30
	Poorer	1,634	18.87
	Middle	1,675	19.34
	Richer	1,738	20.06
	Richest	2,117	24.44
**Family size**	Five or less	4,270	49.28
	Above five members	4,393	50.72
**Under five children**	No	4,387	50.65
	Yes	4,276	49.35
**Age of household head**	15–30 years	2,266	26.15
	31–40 years	2,232	25.76
	41–64 years	2,912	33.62
	65 + years	1,253	14.47
	Mean ± SD = 43.04 ± 16.49 years		
**Health facility visits**	Only one visit	7,717	89.07
	Two or more visits	946	10.93

### Health insurance coverage

From a total of 8663 households participated in the survey, only 2,434 (28.1%) of them were covered by health insurance. About 29.3% of male headed and 24% of female headed households were covered by health insurance. More than half (55%) of households in Amhara region and only 3.5% of households in afar region were covered by health insurance Table 2.

**Table 2:** Health insurance coverage by socio-economic characteristics of study participants

Variables	Health insurance coverage	
	**Not covered**	**Covered**
**Sex of household head**		
Male	4,776 (70.7)	1,975(29.3)
Female	1,452(76)	459 (24)
**Place of residence**		
Urban	2,147(80.6)	517(19.4)
Rural	4,082 (68)	1,917(32)
**Age of household head**		
15–30 years	1,877(82.9)	388(17.1)
31–40 years	1,594(71.4)	638(28.6)
41–64 years	1,924(66)	989(34)
65 + years	834(66.6)	419(33.4)
**Administrative regions**		
Tigray	334(57.2)	250(42.8)
Afar	84 (96.5)	3(3.5)
Amhara	949 (45)	1,160(55)
Oromia	2,588(80.7)	619(19.3)
Somali	402(96.2)	16(3.8)
Benishangul-gumuz	85(90.4)	9(9.6)
SNNP	1,343(80.5)	325(19.5)
Gambela	33(91.7)	3(8.3)
Harari	22(88)	3(12)
Addis Ababa	334(88.8)	42(11.2)
Dire Dawa	53(94.6)	3(3.4)
**Family size**		
less than 5 members	3,146 (73.7)	1,124(26.3
5 and more members	3,083(70.2)	1,310(29.8)
**Wealth index**		
Poorest	1,182(78.9)	316(21.1)
Poorer	1,165(71.2)	470(28.8)
Middle	1,037(61.9)	638(38.1)
Richer	1,135(65.3)	603(34.7)
Richest	1,710(80.8)	406(19.2)

### Factors associated with health insurance coverage

In multivariable logistic regressions administration regions, place of residence, sex of household head, wealth index, Family size, Under-five age children, and Age of the HH head were factors identified as determinants of health insurance coverage.

Households who live in Tigray, Amhara, Oromia, SNNP regions and Addis Ababa city were more likely to be covered by health insurance as compared to those live in Afar Region (Tigray: AOR = 16.9, 95%CI: 5.53, 51.59, Amhara: AOR = 25.8, 95%CI: 8.52, 78.02, Oromia: AOR = 4.27, 95%CI: 1.41, 12.92, SNNP: AOR = 4.06, 95%CI: 1.34, 12.32, Addis Ababa: AOR = 4.65, 95%CI: 1.46, 14.78).

Respondents who were rural residents had 1.38 times higher odds of being covered by health insurance compared to urban residents (AOR = 1.38, 95%CI: 1.17, 1.63). The odds of being covered by health insurance were 1.23 times higher among male headed households compared to female headed households (AOR = 1.23, 95%CI: 1.07, 1.41). Respondents who were from middle and richer wealth index had higher odds of being covered by health insurance compared to those from poorest wealth index (Middle: AOR = 1.75, 95%CI: 1.46, 2.09, Richer: AOR = 1.86, 95%CI: 1.55, 2.24). Household with 5 or more members had 1.17 times higher odds of health insurance coverage compared to those with less than 5 member (AOR = 1.17, 95%CI; 1.029, 1.33). Household having under five years age had 1.22 times higher odds of health insurance coverage (AOR = 1.22, 95%CI; 1.076, 1.38).

The older household heads had higher odds of health insurance coverage than those aged 15–30 years (31–40 years, AOR = 1.71, 95%CI: 1.45, 2.01, 41–64 years, AOR = 2.49, 95%CI: 2.12, 2.92, 65 + years, AOR = 2.43, 95%CI: 2.01, 2.93) (Table 3).

**Table 3:** Multivariable logistic regression of factors associated with health insurance coverage in Ethiopia, 2019

Variables	Covered by health insurance		COR(95% CI)	AOR(95% CI)
	No	Yes		
**Administrative regions**				
Afar	84	3	Ref	Ref
Tigray	335	250	18.98 (6.27, 57.42)	16.9(5.53, 51.59) ^*^
Amhara	949	1,160	30.97 (10.32, 92.89)	25.8(8.52, 78.02) ^*^
Oromia	2,588	619	6.06 (2.02, 18.17)	4.27 (1.41, 12.92) ^*^
Somali	402	17	1.04 (0.31, 3.45)	1.00(0.30, 3.35)
Benishangul-gumuz	85	9	2.68 (0.73, 9.77)	1.91(0.52, 7.04)
SNNP	1,343	325	6.13 (2.03, 18.44)	4.06 (1.34, 12.32) ^*^
Gambella	33	3	2.08 (0.40, 10.91)	2.03 (0.38, 10.85)
Harari	22	4	4.09 (0.85, 19.71)	4.06(0.82, 20.07)
Addis Ababa	334	42	3.17 (1.01, 9.93)	4.65 (1.46, 14.78) ^*^
Dire Dawa	53	3	1.56 (0.33, 7.44)	1.86 (0.38, 8.96)
**Place of residence**				
Urban	2,147	517	Ref	Ref
Rural	4,082	1,917	1.95(1.75, 2.18) ^**^	1.38 (1.17, 1.63) ^**^
**Sex of household head**				
Female	1,453	459	Ref	Ref
Male	4,776	1,975	1.30(1.16, 1.47) ^**^	1.23(1.07, 1.41) ^**^
**Wealth index**				
Poorest	1,181	317	Ref	Ref
Poorer	1,165	470	1.5(1.28, 1.77) ^**^	1.07 (0.89, 1.28)
Middle	1,037	638	2.29(1.96, 2.69) ^**^	1.75 (1.46, 2.09) ^**^
Richer	1,135	603	1.98(1.69, 2.32) ^**^	1.86 (1.55, 2.24) ^**^
Richest	1,710	407	0.89(0.75, 1.04)	0.94(0.75, 1.18)
**Family size**				
< 5 family member	3,146	1,124	Ref	Ref
5 or more family size	3,083	1,310	1.19(1.08, 1.31) ^**^	1.17(1.03, 1.33) ^**^
**Under-five age children**				
No	3,148	1,239	Ref	Ref
Yes	3,080	1,195	0.98 (0.89, 1.08)	1.22(1.076, 1.38) ^**^
**Age of the household head**				
15- 30 years	1,877	388	Ref	Ref
31–40 years	1,594	638	1.93(1.68, 2.23) ^**^	1.71 (1.45, 2.01) ^**^
41–64 years	1,924	989	2.48(2.17, 2.84) ^**^	2.49 (2.12, 2.92) ^**^
65 + years	834	419	2.43(2.07, 2.85) ^**^	2.43 (2.01, 2.93) ^**^
**Health facility visits**				
Only one visit	5,532	2,185	Ref	Ref
Two or more visits	697	249	0.90(0.77, 1.05)	0.88((0.74, 1.05)

^*^ significant at *p*-value < 0.05

^**^ significant at *p*-value < 0.001

## Discussion

The health insurance coverage in Ethiopia is was found to be 28.1% (95%CI: 27.2%, 29%), this figure was lower than the study conducted in Tanzania 49% [17], and Rwanda 85% [18]. This difference may be attributed to differences in implementation strategies, premium payments and benefit packages. This figure is quite low as compared to other studies conducted in pilot districts 52.4% [19], Northwest part of Ethiopia 42% [20], and West Gojam Zone 58% [21].

This study figured out huge difference in the coverage of health among administrative regions ranging from 3.4% in Dire Dawa to 55% in Amhara. After adjusting for other covariates administrative regions independently associated with health insurance coverage. Compared to Afar region, the odds of health insurance coverage was higher in Amhara, Tigray, Oromia, SNNP, and Addis Ababa. This significant difference among administrative regions is mainly attributed to the differences in their economic activities and delay in implementation of the scheme in those low coverage regions. The Ethiopian government launched the community-based health insurance scheme in June 2011 at 13 in piloted districts found in Oromia, Tigray, Amhara, and Southern Nations, Nationalities and Peoples (SNNP) regions [10]. In regions such as Afar, Somali, Benishangul Gumuz, and Gambella the CBHI was launched after 2–3 years of implementation in the other regions [22].

In the current study, the place of residence was a significant contributor of the CBHI scheme utilization. In doing so, we found that, the rural residents were more likely insured as compared to the urban residents. The consistent findings were reported from Senegal [23]. In Ethiopian context the CBHI scheme; the better coverage by rural residents compared to urban residents may be attributed to the primary targets of the CBHI; the insurance type widely implemented in Ethiopia. Moreover, more than two-third of the Ethiopian population reside in the rural setting. However, this finding is in contrast with the study result from Tanzania, which stated that urban communities were more likely insured than the rural [24]. This might be the difference in study setting and types of scheme implemented.

We found that male headed households had higher odds of being covered by health insurance compared to the female-headed household. Previous study findings conducted in Burkina Faso [25] and Nigeria supported the current result [26]. This could be linked with the fact that men are presumed to be responsible for household’s financial decision and a breadwinner in most African countries. In contrast to the current study finding the previous study finding from northern Ethiopia showed that the female-headed households were more likely insured than the male-headed household [26].

Households with a richer and middle wealth index had higher odds of being covered by health insurance compared to poorest households. This is in agreement with existing evidences [16, 27–29]. The affordability of premium may be linked with the enrolment of households with higher wealth index. Moreover, Vialle et al. (2008) suggested that poor households have less enrollment status, which may be attributed to the fear of extra cost during health care utilization like transportation and accommodation cost, which are not covered by the insurance scheme [30].

Being from large family size increased the odds of health insurance coverage as compared to their counterparts. This is in consonance with previously conducted studies in Ethiopia [22, 27], Tanzania [16], Sri-lanka [31] and Nigeria [28]. This could be explained by the fact that, households with a large family size prefer to enroll the health insurance by the logical comparison of the expected premium and expected cost of health care paid out of pocket or the fear of catastrophic health expenditure. In contrast to the current finding studies conducted in Burkina Faso [32] and India [33, 34] revealed that, households with larger family size were more likely to dropout from community based health insurance scheme. In addition to the family size, the current study shows that households having the children aged less than five years old were more likely insured as compared to their counterparts. In agreement to the current finding Abdel_Ghany et al. (2001) reported that those household have a children of less than six years were more likely insured than those does not have a younger children [35]. This could be due to the frequent health care visit of families having younger children.

The current study signified that the age of household head is significantly associated with health insurance coverage. Accordingly, households with older age group were more likely utilize the health insurance as compared to the younger household members. Similarly Bhat et al. (2006) reported that reference households with a higher age were more likely insured [36]. This finding is also consistent with the previous study results [24, 35, 37]. This might be due to the fact that as the age of household head increases the probability of having chronic illness may increase and this could attribute for the need to be insured for better access to health care services.

### Strengths and weakness of study

The study utilized data from a nationwide survey, which could be considered as strength and could enhance representativeness. The limitation is that the cross sectional study design cannot establish temporal relationship between the outcome and response variables. Another limitation of the study is the 2019 mini-EDHS household datasets used in this analysis did not collect data on education, and employment variables. Moreover, health insurance scheme related factors are not included in the study.

## Conclusions

Less than one-third of Ethiopians were covered by health insurance which is far lower than the national targets of covering 80% of the eligible groups by 2020 [13]. Administration regions, place of residence, and wealth index were significantly associated with health insurance coverage. Regional and rural–urban disparities in health insurance coverage call for contextualized implementation strategies. For pastoralists dominated regions such as Afar, Somali, Gambella, and Benishangul Gumuz the government should arrange premium subsidization and employ a wide range of promotion activities. Even though the currently implemented CBHI targets the low income rural majorities, those with higher wealth indices were more likely to be covered by health insurance and this implies the need to more subsidies for poor households. Moreover, male and older age headed household were more likely to be covered by health insurance. The insurance scheme should reconsider its implementation strategies to increase health insurance uptake of the younger and female headed households. Another interesting finding is that those with large family size and under-five children were with higher odds of being covered by health insurance. This should be capitalized as it increases the utilization of primary health care by larger family and under-five children and community mobilizations targeting household of small family size should also be emphasized. In summary, to achieve the national targets of health insurance coverage, implementation strategies that take into account these factors would help increase the insurance coverage.

## Supplementary Information

**Additional file 1.**

## Data Availability

This research uses national survey data that is publicly available for research collected by the Central Statistical Agency of Ethiopia and ICF. The 2019 mini Ethiopian Demographic and Health Survey dataset can be accessed at (http://idhsdata.org) upon reasonable request.
