# Supplementary material for: Health insurance coverage in Ethiopia: financial protection in the Era of sustainable cevelopment goals (SDGs)
Source: Health Econ Rev. 2022 Aug 3;12:43. doi: 10.1186/s13561-022-00389-5 (PMC9347146; doi:10.1186/s13561-022-00389-5)
Supplement: Supplementary file 1 — Additional file 1. [file 13561_2022_389_MOESM1_ESM.docx]

HECR-D-22-00008
Health Insurance Coverage in Ethiopia: Financial Protection in the Era of Sustainable Development Goals (SDGs)
Bedasa Taye Merga; Bikila Balis; Gelana Fekadu

Health Economics Review

**Authors’ Response to reviewer 1 comments**

| **Reviewer 1 comments** | **Authors’ response** |
| --- | --- |
| a) Were survey weights applied? | Thank you. The analyses were employed on weighted data. |
| b) Important socio-economic variables, such as education, employment etc....were omitted. The authors should comment if there were applicable variables for the household (e.g. education / employment of household head) or state as a limitation. | Thank you. The household datasets used in this analysis did not collect data on education, and employment variables. We clearly stated this limitation in the revised manuscript. |
| c) The striking regional differences were not discussed by the authors. I suggest elaborating on geographic differences. | Thank you for your invaluable inputs in improving manuscript. Your comments are all considered critically. Now the authors discussed the regional differences in health insurance achievement so far. |
| d) The recommendation is somewhat general: "to meet the national goal, socio-demographic factors should be considered". I suggest the authors provide some viable suggestions how the survey results could inform national strategies aiming to expand health insurance coverage in Ethiopia. | Thank you. Now the authors revised the conclusion part and recommendations are forwarded based on the findings. |

**Authors’ Response to reviewer 2 comments**

| **Reviewer 2 comments** | **Authors’ response** |
| --- | --- |
| I am not so clear about the odds ratios for the different administrative regions. What does 16.9 for the Tigray region denote when comparing it to the Afar region? | Thank you for your concern. This large odds ratio denotes the significant difference in the proportion of outcome variable between the regions. |
| Despite the increase in support for the expansion of scheme across the pilot districts the recent evidence shows the rate of enrollment is low across the implementation sites indicating that the scheme have [sic] failed to address the intended groups of the populations.  This might be linked with the fact that the suggested financial contributions are regardless of the socioeconomic status and regional differences." (p.13) This paragraph actually points towards the major weakness of the study, i.e. in that it remains at the purely descriptive level. The key question is, how come that despite increased support for health insurance, certain populations are less well reached. Whereas the effort to reach the rural populations seem to work as the rural areas are better covered, other efforts do not seem to be effective, e.g. reaching out to female heads, younger household heads, and the poor. | Thank you for your critical comments that helped us in improving our manuscript. The study figured out the health insurance coverage at national level and established factors associated with the coverage. As you rose some factors from the supply side or scheme related factors are not included in the study. And we included in the weakness of the study.  In Ethiopia, CBHI is the health insurance type which is currently implemented in most of rural areas and mainly targeting low income markets such as those engaged in agriculture sector. That is why rural residents are more covered than urban residents. This is clearly stated in the revised manuscript. |
| The authors write in their conclusions (p. 16): "were found to determine the health insurance coverage in Ethiopia." This is too strongly formulated, probably better would be "… were found to be correlated with …" | Thank you for critical review and constructive comments. The statement is rewritten using appropriate terms. |
| The authors conclude "Therefore, devising strategies that take into account socio-economic status and demographic factors of the eligible group would help increase the insurance coverage." This border on the obvious and I would like to see far more concrete implications for policy-makers. | Thank you very much. Now in the revised manuscript the conclusion part is thoroughly revised and based on the finding recommendations are forwarded. |
| What to make of the finding that richer, larger households with a male elderly head are more likely to take up health insurance. Why is this case? How to translate the findings into a national policy aiming at wider coverage? | Thank you. The policy implications of the finding are discussed under discussion and conclusion part. |
| The huge difference between the administrative regions point to operation, administrative and logistical issues in promoting the use of social health insurance schemes. The regional CBHI schemes - are they all the same or do substantial differences exist between the schemes? The large differences point towards substantial differences. | Thank you for your invaluable inputs in improving manuscript. Your comments are all considered critically. Now the authors discussed the regional differences in health insurance achievement so far. |
| Table 1: it would be most useful to have the corresponding data for Ethiopia added, as this would allow for an assessment of the representativeness of the study. | Thank you. In the table 1 title we included the sample size which allows assessing the representativeness of the data. Moreover, the data utilized in the study is publicly available up on reasonable request. |
| The distribution amongst the wealth quintiles is somewhat skewed with the richest quintile significantly over-represented - this is not discussed in the study. | Thank you. We have checked for the distribution amongst wealth quintiles and found insignificant skewedness. |
| What does “In favor of the argument,” (p.13) mean? | Thank you. We revised the statement. |
| "This is inconsonance with …" (p. 15) should read "in consonance" | Thank you for your critical comments. Now the authors edited the type error. |
